# Increased influence and collaboration: a qualitative study of patients’ experiences of community treatment orders within an assertive community treatment setting

**DOI:** 10.1186/s12913-015-1083-x

**Published:** 2015-09-23

**Authors:** Hanne Kilen Stuen, Jorun Rugkåsa, Anne Landheim, Rolf Wynn

**Affiliations:** Norwegian National Advisory Unit on Concurrent Substance Abuse and Mental Health Disorders, Innlandet Hospital Trust, Brummundal, Norway; Department of Clinical Medicine, Faculty of Health Sciences, UiT – The Arctic University of Norway, Tromsø, Norway; Health Services Research Unit, Akershus University Hospital, Lørenskog, Norway; Departement of Psychiatry, University of Oxford, Oxford, UK; Norwegian Centre for Addiction Research, University of Oslo, Oslo, Norway; Divison of Mental Health and Addictions, University Hospital of North Norway, Tromsø, Norway

## Abstract

**Background:**

Since 2009, 14 assertive community treatment (ACT) teams have started up in Norway. Over 30 % of the patients treated by the ACT teams were subject to community treatment orders (CTOs) at intake. CTOs are legal mechanisms to secure treatment adherence for patients with severe mental illness. Little is known about patients’ views and experiences of CTOs within an ACT context.

**Methods:**

The study was based on qualitative in depth interviews with 15 patients that were followed up by ACT teams and that were currently subjected to CTOs. The data were analyzed by using a modified grounded theory approach.

**Results:**

While some participants experienced the CTO as a security net and as an important factor for staying well, others described the CTO as a social control mechanism and as a violation of their autonomy. Although experiencing difficulties and tensions, many participants described the ACT team as a different mental health arena from what they had known before, with another frame of interaction. Despite being legally compelled to receive treatment, many participants talked about how the ACT teams focused on addressing unmet needs, the management of future crises, and finding solutions to daily life problems. Assistance with housing and finances, reduced social isolation, and being able to seek help voluntarily were positive outcomes emphasized by many patients.

**Discussion:**

The participants had different views of being on a CTO within an ACT setting. While some remained clearly negative to the CTO, others described a gradual transition toward regarding the CTO as an acceptablesolution as they gained experience of ACT. Many of the participants valued the supportive relationship withthe ACT team, and communication with the care providers and the care providers’ attitudes could make a significant difference. The study shows that the perception of coercion is context dependent, and that the relationship between care providers and patients is of importance to how patients interpret the providers’ behavior and the restrictive interventions.

**Conclusions:**

Although some patients focused on loss of autonomy and being compelled to take medications, other patients emphasised the supportive relationships they had with the ACT teams and that they had received help with housing, finances, and other daily life problems. Thus, being on mandated community treatment could be acceptable in the opinion of several of the patients, provided that they received other services that they found beneficial.

## Background

Use of compulsory admissions and community treatment orders (CTOs) are by international standards, high in Norway [1–3]. The use of coercion raises a range of ethical, clinical, and legal questions [4]. Reducing the use of coercion within mental health care settings has been given high priority in government policy, and in 2006 a national action plan was introduced. Even so, there has been little debate about CTOs in Norway, which have been used since the Norwegian Mental Health Act (MHA) [5] was implemented in 1961. CTOs are legal interventions to enhance treatment compliance for persons with severe mental illness that have a history of disengagement from services and frequent re-admissions. Besides mandating patients to attend appointments and adhere to treatment, the CTO regime also relies on the provision for rapid recall to hospital for its enforcement. Since 2001, as part of an amendment of the MHA [5], CTOs can also be established in an outpatient setting.

In the Norwegian legislation, the need for compulsory admission and CTO is regarded as a clinical decision, and it is based upon the responsible psychiatrist’s or clinical psychologist’s judgment. It does not require a court verdict, but the decision may be appealed to a Supervisory Commission that is headed by a judge. The decisions of the Supervisory Commission may be appealed to the civil court system. The legal criteria for compulsory admissions and treatment are that the patient suffers from a severe mental disorder and that the possibility of cure or considerable improvement will be lost if the patient is not admitted or treated, or that treatment is deemed necessary to prevent harm to self or others [6]. Unlike most other CTO regimes, in Norway, a separate Medication Order is needed to compel patients to take medication. If the patient subjected to a CTO and a medication order refuses medication, the patient may be taken to a psychiatric facility to receive medication involuntarily there [5]. The responsible clinician is obliged to conduct a clinical assessment every third month to uphold the CTO. The Supervisory Commission controls all relevant documents and performs independent reviews to decide whether the legal conditions of the CTO are fulfilled.

While there has been much discussion internationally regarding the effectiveness of CTOs [7–9], it has been estimated that above 30 % of the involuntarily admitted patients in Norway are discharged onto CTOs [10]. Moreover, there was an estimated 50 % increase in the use of CTOs between 2002 and 2007 [11]. CTOs were first explicitly mentioned as an integrated part of government policy in a white paper from 2012, where increased voluntariness and patients’ autonomy were presented as guiding principles for mental health care [12]. In this policy document, ACT was a recommended treatment model for individuals with severe mental illness, a history of treatment refusal, and frequent relapses, with the aim of reducing the need for involuntary hospital admissions [12].

The Norwegian Directorate of Health financially supports the implementation of ACT in Norway and since 2009, 14 ACT teams have started up. This qualitative study formed part of a national evaluation of the implementation of ACT teams in Norway. ACT has been described as a robust model of community-based treatment for patients with severe mental illness with complex needs, who have not benefitted from traditional health services. ACT teams provide continued and comprehensive mental health and social rehabilitation services, and offer support and practical assistance with daily life in the community. A Cochrane review concluded that ACT compared to standard mental health care reduces frequency and length of hospital admissions and improves housing stability, employment, and results in higher patient satisfaction with services [13]. Besides increased medication adherence and improving patients’ social functioning, social inclusion and recovery are important aims of ACT [14–16].

ACT teams are typically multi-disciplinary, and usually include a psychiatrist. Team members share responsibility for the patients. The teams have a low caseload and frequent team meetings, in order to be able to follow up patients closely. The low caseload ensures that ACT teams can offer intensive services and respond flexibly and proactively during crises. Teams have a ‘no discharge’ policy. ACT teams typically use specific engagement techniques, such as assertive outreach and providing practical help and assistance with daily life problems such as housing and patients’ finances, in order to comprehensively meet patients’ needs [17–20].

There are also some studies that examine CTOs, although most of these have not examined patients in ACT. It has been suggested that CTOs can improve outcomes if sustained over time (for more than six months) and if linked to intensive services. However, there has been much debate about these findings [21–24]. Prior studies that have examined patients’ experiences with CTOs have pointed to certain topics as particularly central, such as loss of autonomy and being pressured to take medications, while other studies have emphasised perceived benefits such as increased social functioning [25–28]. The aim of this study is to obtain more knowledge about how patients experience CTOs within an ACT context. The study aims to shed light on patients’ experiences with informal and formal strategies used to promote continued treatment engagement, and to gain insight into how CTOs impact their daily lives.

## Methods

### Design

The study draws on a modified grounded theory approach [29], inspired by a constructivistic and interpretative framework, which is a recognized method for investigating phenomena where limited prior knowledge exists. An inductive approach enables depth and richness of data generation, and facilitates the description and development of a conceptual understanding in terms of social actors’ motives, actions and accounts. Qualitative interviews [30, 31] were used to examine the patients’ experiences. Participants were sampled purposively from four ACT teams that varied in size and in their use of CTOs. An iterative process of data collection and analysis, inspired by Charmaz’ flexible guidelines [29], was used to explore the complexity of individual experience and perceptions.

### Recruitment and sample

Purposive sampling methods [32] were used to gather rich data, based on a maximum variation sample. Two urban and two rural ACT teams were selected. We first contacted the team-leaders to inform about the study 30 months after the teams started up. At this stage, one of the urban teams had 76 included patients, of which 40 were on a CTO. The other urban team had 68 patients, of which 23 were on a CTO. One of the rural teams had 67 patients, of which 4 were on a CTO. The other rural team had 38 patients, of which 13 were on a CTO. The team leaders were given written information about the study and a separate information letter that was forwarded to the patients. The inclusion criteria in this study were that the participants had been included in the ACT-team, and been subjected to a CTO for at least six months. Patients that the ACT teams deemed to ill to participate were excluded. To secure rich and nuanced accounts, positive as well as negative, we emphasized that we wanted to include males and females, with and without a compulsory medication order and persons with and without co-ocurring substance abuse problems. The team leaders decided which staff members that made the first approach to the patients to ask if they were willing to participate in the study. We recruited new participants until data saturation occurred and subsequent interviews did not reveal substantial new information.

### Interviews

Data were collected by the first author (HKS) through in depth qualitative interviews in the period from September 2013 to February 2014. In three interviews, a staff-member introduced HKS as an interviewer, and in two of these they left before the interview started. In the third interview, the staff-member stayed for the first ten minutes and left when the participant was comfortable to continue the interview without his presence. Because this interview lasted for 90 min, we do not believe the staff-member’s initial presence affected the participant’s responses. A total of 15 interviews were conducted (see Table 1 for an overview of the characteristics of the participants). Nine interviews were conducted in the participants’ homes, while six participants preferred to have the interview somewhere else (i.e. at the outpatient clinic, at the ACT-teams’ facilities, etc.).

**Table 1:** Characteristics of sample

Gender	Male	9
	Female	6
Age	<37	5
	37–47	6
	>47	4
Diagnoses	Psychotic disorders, including schizophrenia	10
	Schizoaffective disorder	5
Active substance abuse	Yes	9
	No	6
Accomodation	Independent apartment	9
	Supervised apartment	6
CTO duration (current)	Less than 1 year	4
	More than 1 year	11
Medication order	Yes	4
	No	11
Antipsychotics	Depot	12
	Oral	2
	None	1

A thematic interview guide was developed jointly by the authors, based on a literature review and a pilot interview with one patient. All the interviews started with open questions about the participants’ everyday lives and whom they had to rely on if they needed someone to talk with or help them solve problems. We continued to ask more specific questions about why and how the CTOs were established, about the content of the CTOs and how the CTOs influenced on their daily lives. We followed up with clarifying questions. After the first interviews, one central theme that emerged in the participants’ accounts was their equivocal perception of CTOs and the ACT team’s enactment of the order, and the association between coercion and motivation, which we followed up and explored in depth in the following interviews. The interviews lasted from 35 to 120 min, and all but two were recorded digitally and transcribed verbatim. These two patients did not want to be recorded, but accepted that detailed notes were taken.

### Analysis

An iterative process of data collection and analysis was used to develop a conceptual understanding of the participants’ experiences, actions and strategies, based on main categories grounded in the data [29]. The first eight interviews were conducted within a concentrated time period because of long travelling distances. The interviews were first listened through several times, and reflections and thematic codes were written down. Then the first eight interviews were transcribed verbatim, and the initial coding identified meaning units in the transcribed text. After the initial coding, we compared the most frequently used codes by going through larger segments of data, in order to develop more focused codes. Subsequently, we followed the same procedures with the other interviews. After the initial coding, the most central codes were clustered in theoretically linked themes, to develop categories and sub-categories, based on properties and dimensions (focused coding). Subsequently, categories were linked together (theoretical coding). The initial and focused coding was done manually, and thereafter the data was moved into the NVivo software [33]. The process of constant comparative analysis was continued with the help of the software until no additional new observations or information emerged (i.e. when data saturation was reached). Memo writing was used through the process, to increase the level of abstraction and to develop the categories.

### Ethical considerations

The study was approved by the Regional Committee for Medical and Health Research Ethics, South-East Region (case file number 1196/2010). All participants were informed that their participation was voluntary and that they could withdraw their consent at any time. Written informed consent was obtained after HKS assured that the participants were informed about the purpose of the study. There was an agreement with the ACT teams that the participants could contact them after the interview if the interview elevated emotional difficulties. Many of the participants experienced not to have a choice regarding medications, and the compulsory nature of treatment was a key theme. Medication monitoring is an important component within ACT, together with a wider psychosocial approach to meet the patients’ complex needs. Use of involuntary treatment and medications is a contentious issue and it is therefore important to clarify that we believe medications may be beneficial for some patients and less beneficial or even unneccesary for other patients. We believe the circumstances of each individual patient must be taken into consideration when considering the matter. However, while we acknowledge that our view might have impacted the interpretation of the data, we aimed to explore the patients’ own views and experiences.

## Results

In our analysis, we identified three main categories that reflected a tension between the perceived restrictions of the CTO on the one hand and receiving care, treatment and coordinated services from the ACT team on the other (Table 2). First, we describe the patients’ experiences with involuntary treatment, second the importance of therapeutic relationships with the ACT team, and third, the patients’ understanding and strategies, with regard to collaboration with the ACT team.

**Table 2:** Main categories and sub-categories

Main categories	Sub-categories
Experiences with involuntary treatment	Control and protection
	Lack of influence on medication
	Coercion context
The importance of trusting relationships	Building trust
	A frame for interaction
	Negotiations about treatment
Collaboration as strategy	Reflecting on the need for treatment and care
	Security net

### Experiences with involuntary treatment

Most participants were under a CTO with prescribed antipsychotic depot medication when they were admitted to the ACT-team. The purpose of the CTO was to ensure continued treatment after discharge from an involuntary hospital admission. Although reporting that they were obliged to have regular contact with the ACT-team, most participants described the coercive elements of the CTO to be that they had to take medications and that the psychiatrist had the authority to impose restrictions.

### Control and protection

Most patients explained the CTO by their refusal to take medications:

”*I do not want to take antipsychotic medications. That’s why I’m under a CTO.”*

Only one participant reported additional conditions attached to the CTO beside medications and appointment attendance. In addition to having a guardian and being followed up by the ACT team under a medication order, she had to live in a supervised residency as part of the CTO. She was not happy with the arrangement:

*“I didn’t realize that it would be like this. I feel stigmatized. My whole life has been taken over, controlled by others.*”

Like some of the other participants, she described the CTO as a legal mechanism to monitor and control her behaviour, and as a violation of her civil rights and autonomy. Some participants described the CTO as part of an integrated sequence of coercive interventions based on persuasion, pressure, threats, and use of force as a means of control. One participant said:

“*I’m a bit paranoid, but that’s no reason for keeping me under detention for more than ten years. They want to have me in the system to have me under control.”*

Some of the other participants, who described symptom reduction and improved life conditions, regarded the CTO more as a protective framework, and as a positive component in their recovery. As one participant said:

“*It is to hold me, so that I won’t use substances early in my recovery process.”*

Another patient, who recently had been discharged and now was on his first CTO, emphasized:

”*The reason why they put me on a CTO was to secure my legal rights and to ascertain that I would adjust.”*

Like some of the other participants, he regarded the CTO as a legal tool to prevent relapse. He referred to the simplified admission routines, which enhanced sustained follow-up, free medications, and free dental treatment – all components of the patient’s CTO.

### Lack of influence on medication

The participants’ views and experiences of the CTO was often influenced by previous negative experiences from the mental health services. They not only addressed the legal authority of clinicians and coercive practices, but also how they encountered a strict medical understanding where their opinions, judgements and wishes were ignored. A perceived overemphasis on medications, a lack of clarity and predictability and a lack of involvement and respect were the most prominent points the patients made. Many of the participants had been under different coercive regimes for many years. They had been mandated to use medications they often did not believe that they needed, and which they often felt had more negative side effects than benefits. Some made the point that the medications made them numb, tired and passive, and several stated that medications did not eliminate their auditory hallucinations or delusions. CTOs are often used for sustained periods of time, and the uncertainty about the duration of the CTO was difficult to accept for several of the patients:

“*The psychiatrists believe I don’t know my own best interest after 42 years. It is just like I am a big idiot. I have tried antipsychotic medications for 15 years, so I should have some idea of what I am talking about. It’s like talking to deaf ears.”*

Several participants wanted to have the CTO discontinued in order to come off medications completely. Other participants questioned the psychiatrists’ insistence regarding the continued use of depot antipsychotics:

“*To be compelled to use depot medication in an indefinite time period is the worst of it, it takes away all my motivation and hope for the future. I have managed by using little medication, which I have administrated on my own. I have managed fine. I can need an admission in life crises. I am being overmedicated, and that depot makes me unwell.”*

The control assessments often focused on previous illness episodes, and some of the patients felt they had few opportunities to talk in depth about their daily life and their thoughts about the future. The patients described that previous crises were the most important factor with respect to how their present life situation was understood. Typical statements from the patients were:

“*Everything is being documented and is being used against me*”

“*I am not being heard*”

In these cases, giving the patient medication was typically seen as the only solution by the health professionals. The experience of being in a confined position, without influence on treatment decisions was perceived of as disempowering by the patients, as was encountering low expectations about their future prospects.

### Coercion context

For many participants, the initial contact with the ACT-team had been established in the hospital ward, where the same ACT-team members had picked them up when they were discharged from the hospital and helped them to get settled in their apartments. Although being compelled to use medications, many participants valued the ACT teams’ comprehensive and coordinated services. Many of the participants used phrases like:

”*There is not much coercion”* and *“It is not voluntary, but it is not coercion either.”*

Most of the participants described the CTO as compelling them to take the prescribed medication and to accept the psychiatrists’ authority to impose restrictions. When being asked about the consequences of treatment refusal, many referred to the potential threat of being readmitted to hospital, and not least, the realistic prospects of a medication order:

*“I have to use medicines whether I want to or not, but it will end up with more coercion and it will be more difficult if I am not willing to take the injection.”*

Another participant said:

*”That would have had consequences, and then they would come after me. Then there would be coercion.”*

One participant claimed that the ACT team had not initially informed him that the CTO that was in effect did not authorize compulsory treatment. When he refused to take antipsychotics, the psychiatrist had initiated a compulsory medication order, which the County Medical Officer then rejected because of a formal mistake. He had recently been informed that a medication order had now been established according to the proper procedures:

*“There is a threat behind. There is no point to take it further. I’m not being heard anyway. Both the supervisory commission and the County Medical Officer do as the physician recommends. We have no say. They believe it is important that I use medications. It has a high price. I feel that it is for the safety of the system.”*

Many participants described treatment pressure as an integrated part of mental health care, and that coercion did not depend on a CTO. The participants’ accounts illustrate the blurred distinction between formal and informal coercion by referring to the responsible clinician’s discretionary powers:

*“If I had been transferred to a voluntary status I would have to use medications anyway. If you accept taking medications, the legal compulsion will be discontinued, but if you refuse, if you don’t want to take medications, you will be put back on a CTO. There is coercion regardless whether you have a voluntary or involuntary legal status…You don’t have the right to choose how you want to live your life or which medications you want you use, like a normal person…The more they counteract me, the more I counteract them.”*

While the patient described the consequences of coercion as distrust and reactance, he discussed having been given a new treating psychiatrist as a potential turning point:

*“At least I am in a dialogue with her. She recognized that the psychosis could be caused by other things… Then I was more receptive. She has been right in some of the things she has said, she is at least forthcoming, and she tries to understand and to do something about the situation.”*

### The importance of trusting relationships

Many of the participants who had long histories of being under coercion described a gradual transition from resistance to medication toward developing a therapeutic alliance with the ACT-teams, based on an ongoing dialogue about how to address unmet needs and future goals. Although being compelled to use medications, many described the ACT team’s availability, practical support and assistance in solving daily life problems. They also emphasised that they were being listened to. The patients described the uniqueness of being treated by an ACT-team, and especially that the frame of interaction differed from other types of mental health treatment.

### Building trust

Several emphasized the importance of taking small steps and that building trust takes time:

“*There is something special when you have your own apartment. They enter your private area. It has to be someone you know, that you have built a relation to, who you trust…building trust takes time.”*

In addition to the practical and emotional support they felt they received from the ACT-team, many valued the team’s availability. Several mentioned that it was important for them that they had someone they could call for practical help or for social contact. While some spoke of the team as a whole, others referred to specific team-members who “walked that extra mile” to find acceptable solutions. As one participant said, by referring to the helpfulness and kindness of a social worker in one of the teams:

“*I don’t know what I should have done without X*.”

One participant who had been in care of the team for six months emphasized:

*“I haven’t talked to them about personal stuff yet, but she (the psychiatrist) said it would take time to get to know each other, and that it will not happen at once. They just take me as I am. That’s nice. The most important is that they pick up the phone when I call, and to know that they are accessible when I call. They have helped me with lots of things.”*

Despite having weekly medication delivery appointments she said:

“*I contact them. I’m able to wash and make food and stuff. I can be alone most of the time, there is no fuss. I wouldn’t have put up with that*.”

Being able to live their lives in their own apartments and having appointed a guardian to safeguard their finances was described as important life improvements. “At least it’s a start” was how one participant who previously had stayed with friends or in shelters, who recently had moved into a supervised residency, described his situation.

### A frame for interaction

Many participants had difficult experiences from mental health care, and were struggling with personal losses, distress and lack of engagement. The dreary everyday life and lack of joy were explained as the main reason for their wish to have the CTO discontinued to come off medications. As one participant said:

” *I regard myself as part of the community, but I am simultaneously afraid because others are making progress while I have stagnated…Now I am 34 years, I feel full, old and fed up. My experience is that the medications are hindering me. I find it difficult. She (the psychiatrist) is trying to comfort me, simultaneously as her words are forcing me. She wants to collaborate. It is uncertain if I can trust her…I am willing to collaborate with the ACT-team. I realize that they want to help. I am trying to accept that I need medications. I see that the choices I make are of importance.”*

What many of the participants valued most was that staff-members participated in meaningful dialogues, and that they were willing to listen to their experiences and viewpoints. Typical phrases were “*the ACT team talks to us*” and “*the ACT team wants to help.*” Many participants emphasized the importance of having someone who stood up for them, who also coordinated services and followed them through progress and setbacks without being judgmental. Conversations about previous re-admissions, risk situations, how to handle future crises and to find reasonable solutions was described as a fine balance. And as one participant said:

”*It’s important that they* (the ACT team*) have faith in me when I say that I am doing well, that they have confidence in me. They understand the situation, they have the experience.”*

### Negotiations about treatment

Nearly all the participants had weekly contact with the ACT team. While a few participants refused to talk with the psychiatrist other than at the obligatory control assessments, most participants highlighted the importance of being in a dialogue with the psychiatrist about treatment. One participant who recently had discontinued antipsychotic medication without informing the team said:

”*I just felt empty, I just sat there and had nothing to say.”*

When the team found out, the psychiatrist had talked about the risks of the participant stopping taking the medication and informed him that the team would conduct more frequent home-visits to monitor his mental health condition. A few other participants who had tried medication free periods under the CTO had experienced to be re-admitted to hospital, earlier than necessary from their point of view, to start back on medications because of symptom exacerbation. Although the ACT psychiatrist insisted on the need to use medications, many participants emphasized that the psychiatrist had involved them more than they had experienced before, and asked which type of antipsychotic medication they preferred and which dosage they found appropriate. And not least, the psychiatrist had been willing to make a reduction plan and slowly decrease the dosage.

While highlighting the importance of being in a dialogue about medications, many participants stated that medications alone were not sufficient to change their life. One participant said:

“*Individuals who are compulsory detained should receive optimal treatment and follow up, not just sit somewhere not doing anything, that’s not treatment.”*

In addition to basic needs and continued follow-up, increased safety and predictability, were described as important building blocks to have increased control over their life. To have someone to talk with, who followed them though progress and setbacks, was described as an important factor for staying well. As one participant said:

*“It is important that I have contact with the ACT-team. It helps to have someone to talk with and to receive help to learn positive thinking, to be among people with healthy thoughts. I have talked with the psychologist, I talk with X (psychiatrist) once in a while, and I have participated in a group based education session about psychosis. Now I have most contact with two nurses.”*

Besides medication monitoring and providing emotional and practical support, many participants had gradually been encouraged to participate in ACT-organized activities; in-door soccer training, swimming, field trips, golf or activities in the community, educational courses, and voluntary work. And what many valued most were the benefits of receiving coordinated services and assistance with ordinary daily life activities and everyday problems.

### Collaboration as strategy

Many participants were critical of the CTO regime, partly based on previous experiences from mental health services. Being a psychiatric patient was described as a locked position, where they encountered low expectations and pessimistic attitudes. The participants described a process of gradually engaging with the team, weighing various costs and benefits of receiving continued care and treatment under the CTO.

### Reflecting on the need for treatment and care

Many of the participants were still living chaotic lives and many acknowledged the need for help and assistance. One participant who recently had been informed by the ACT psychiatrist that the CTO would not be extended as long as he continued to collaborate and engage in treatment, described the implications of the CTO as: *“A way to survive or handle life.”* And as he emphasized: *“It is all about medications, whether they are good for me or not.”* Even if he was ambivalent about medications, he acknowledged that his family reacted to his behaviour when he stopped taking them and he associated previous admissions with medication discontinuation. And as he said: “*It’s more about functioning in daily life.*” Moreover, he stated that:

*“I have a formative idea that I’ll manage without medications even though everyone else says that I don’t. Now it has been proved several times, but I am not able to realize it and that’s why I am on a CTO. I experience the ACT team as a good place to be and as good people to use.”*

After his last admission to hospital, he and the psychiatrist had met several times to come to an agreement about medications. And, as he emphasized, he had to collaboarate about prescribed treatment, whether the CTO was lifted or not, to get back his driving license. The psychiatrist had agreed to discontinue one of the antipsychotics that had given him bothersome side effects. He stated:

*“I’m still taking Cisordinol, which I guess I have accepted. I have some inner peace…so it might work… I have accepted that I can live with that medication…Now we have agreed that she (referring to the dialogue with the ACT psychiatrist), will support me to regain my driving lisence … and also, practically, they (the ACT team) helped med to clean up my appartement.”*

A few of the participants consistently claimed that they wanted to have the CTO discontinued to come off medications and to avoid contact with mental helath providers. Others stated that they had gradually acknowledged that long term follow up from the ACT team and antipsychotic medication was helping them to manage severe and disabling symptoms. They also often emphasised the importance of ongoing efforts to reduce the side effects of the medications, to improve their daily functioning. A typhical phrase spoken by this group of service users was:*”I want to collaborate with the ACT team.”* One participant explained his grounds for accepting treatment by saying:

*“I have won my civil rights in other matters than the depot injections. That means that I don’t have to be here (at the local psychiatric hospital), and that I don’t have to be locked up. That’s why I have accepted it and collaborate… I chose to collaborate to have increased freedom.”*

While some participants had regularly scheduled admissions at the District Psychiatric Centre, others asked the ACT team to arrange shorter voluntary admissions, as part of their crises plan. One participant tended to ask for shorter inpatient stays after cycles of extensive substance abuse, poor nutrition, and lack of sleep:

”*It is when I have lived too hard.”*

Others described physical health problems such as injection wounds and HIV. Several explained their use of substances, first of all injecting amphetamine, as a strategy to overcome the negative side effects of the medications:

”*It is to regain energy. I become so self-suffering and I stay in bed.*”

Although describing increased housing stability, security and predictability, several still experienced to be “trapped” in a situation with persistent psychiatric symptoms, intolerable side effects, and ongoing substance abuse. Discontinuing antipsychotic medication was by some described as a necessary condition to reduce their substance use. Nevertheless, many participants described the CTO as the least-worst solution, with a restricted set of options and uncertainty about how life would be if they had not been mandated to stay in treatment. Like one participant said, by referring to improved nutrition, more routines and structure:

“*I handle the medications better, and I assume I wouldn’t have made as many life improvements”*

Another participant said:

“*I think it is time to reduce the Fluanxol dosage. I have a low dosage indeed, and I could fall back into psychosis.*”

And for them, as for others, the CTO had been important to facilitate appropriate mental health care and social welfare services they otherwise would not have had access to.

### Security net

By comparing their current life situation to the past, several of the patients made the point that the benefits of receiving supportive and coordinated services from the ACT team overshadowed the disadvantages. Several emphasized that there had been an overall reduction in coercive crises interventions after they had begun receiving services from the ACT team, and as one patient said:

“*The police have often kicked me into the emergency room for a clinical assessment. I don’t know how many times I have been restrained and had injections with medication. Fortunately that’s a long time ago.”*

Like some of the other participants, he was in need of shorter inpatient stays, as he had extensive substance abuse and fluctuating illness episodes. The ACT team often intervened before he reached the point of aggressive behaviour or acute illness. Some participants referred to the CTO as a negotiated agreement and as a necessary safety net to prevent relapse. One participant said:

*“It (the CTO) is in place because I previously have stopped using medications, and I can’t discontinue treatment until I’m well enough to manage at home. It’s positive to know that the team can have me admitted when they see symptoms, and that I can’t refuse. When I’m in a psychosis I can refuse taking medications and resist inpatient treatment even though I need it*.”

Soon after her first CTO had expired she had experienced a serious illness episode when she discontinued medications and refused to have contact with the ACT team. Like other participants she had needed time to accept the need for ongoing medication monitoring and supervision. She needed to have ongoing discussions with the team psychiatrist and the staff-members to figure out the implications of the CTO. The most important difference between the ACT teams and traditional mental health services was the ongoing dialogue about the use of medications:

“*Even if she makes the decisions, I can influence the treatment decisions.*”

## Discussion

One main finding in this study is that the participants had different experiences of being subjected to a CTO while in ACT. While some participants had shorter illness durations and were currently discharged on their first CTOs, others had experienced longer involuntary admissions or been under coercive treatment regimes for most of their adult lives. Although some participants that actively refused treatment were under a medication order, most participants had adjusted to the CTO conditions. In line with Sjöström’s etnographic study [34], several of the participants claimed that they would have to follow recommended treatment whether or not the CTO expired. Some participants who told about ongoing conflicts about the use of antipsychotic medication experienced the CTO as more coercive, based on use of pressure and threats of police assistance for depot medication administration. The medication side effects and the uncertainty about the length of CTOs were described by some participants as the most difficult to accept. Nevertheless, the experience of being legally compelled to receive treatment was often blended with the advantages of receiving other types of care and comprehensive follow-up services from the ACT team. The participants expressed different experiences and opinions. While some remained clearly negative to the CTO, other participants in our study described a gradual transition toward regarding the CTO as an acceptable solution as they gained experience of ACT.

While some participants had experienced longer involuntary admissions, others had previously experienced frequent encounters with the police, repeated emergency room visits and involuntary admissions, often with little social support and follow up after discharge. Many of the participants had lived chaotic lives with active substance abuse. Although Norway is a rights-based welfare state with a well funded health system, the mental health services are fragmented and sometimes poorly coordinated between specialist health care services and primary care services provided in the municipalities [35, 36].

While we are not aware of any prior Norwegian studies of CTOs within an ACT context, two recent qualitative studies explore patients’ subjective experiences of CTOs. Riley et al. [26] found that most of the patients had adjusted to the CTO, which the patients regarded as a less restrictive solution than hospitalisation. The other study showed that most of the patients contrasted the CTO to living an ordinary community life, and the patients felt that the CTO hindered them from activating their own strengths and resources [37].

Many of the patients in our study acknowledged that the CTOs provided access to help and assistance, increased safety and security, as pointed out by Riley et al. [26]. However, some of the patients that described the CTO as a social control mechanism felt that they were not being listened to and treated with sufficient respect, and felt there was a too strong emphasis on medications. This may impede some patients’ self-efficacy and recovery-processes [37, 38].

Even if many participants in our study did not experience that they had a choice regarding medications, few described the ACT team’s enactment of the CTO as disempowering and as a barrier to improvement. Many participants in our study described the ACT team as a different type of mental health service compared to what they had experienced before, with a different frame of interaction. The participants emphasized the teams’ willingness to listen to their views and to have time to reflect on different alternatives, regarding medication as well as basic needs and daily life activities, and that the ACT team understood that medications alone were not sufficient to change the patients’ lives.

The ACT teams’ availability, the flexible combination of interventions, and being offered time, continued care, support and choices were described as the most important improvements compared to traditional services. What many participants valued most was to have someone to call, who acted out of concern for their well being as well as the teams’ willingness to participate in meaningful dialogues. Assistance with housing problems, finances and gradually being encouraged to participate in activities and (voluntary) work were described as important life improvements by many. Others emphasised an overall reduction of coercive crises interventions, more voluntary help seeking, and shorter inpatient stays as important building blocks to gain increased control over their lives. Many of the participants in our study described contrained choices, rather than actual compulsion, and even if extrinsically motivated, many had gradually acknowledged the benefits of receiving continued care, treatment, and supervision under the CTO.

The founders of ACT stated that coercion is not part of the model [39]. Even so, newer model revisions acknowledge that therapeutic limit setting and coercive interventions may be used to safeguard extrinsic motivation for receiving services deemed necessary for continued community living [18]. While some opponents describe CTOs as intrusive and counterproductive to patients’ recovery processes, others justify involuntary treatment as a necessary tool, to be carefully imposed to ensure the right to safe and effective care and treatment [40].

From a patient perspective, recovery is often described as a personal journey, to get one‘s life back on track after the onset of illness. Symptom relief often has a central role in recovery, as well as combined efforts to enable hope, self-determination, personal growth, and responsibility, with the need for creating choices and opportunities for social integration. Although the ACT model has a strong focus on recovery, medication management and monitoring are cornerstones of the model, which has been criticized for being paternalistic and coercive. ACT teams are supposed to continue to engage reluctant persons for up to a year. One question that has been raised by some is whether treatment that cannot be refused is ethically justifiable [41, 42]. While some have claimed that the ACT model is inherently coercive, others disagree [17, 19, 43–46]. Studies have reported that ACT providers sometimes use assertive and controlling techniques. However, supportive relationships and interpersonal influence have been described as the most frequently used mechanisms for promoting treatment goals [43, 47–49]. As some other studies of ACT show, most participants in our study reported that motivational interventions, persuasion, verbal guidance, and education were more frequently used than threats and force [43, 47–49].

Coercion is simultaneously an objective and subjective concept. There may be a blurred distinction between nondirective discussions and coercion. Several of the participants stated that they did not feel coerced despite being subjected to a CTO.

Although being aware of the potential threat of being brought back to hospital or receiving a medication order if they refused treatment, most participants did not describe the ACT team’s enactment of the CTO as coercive. While some participants used phrases like “*I do not experience much coercion*” and “*it is not voluntary, but it’s not coercion either,*” others described the CTO as a negotiated agreement and that they were being “*voluntarily coerced.*”

Motivation is often presented as a critical factor for treatment participation, retention and success [50]. One argument often used is that coerced treatment is likely to fail because the individual does not have an internal motivation. Within self-determination theory motivation is described as a continuum, with amotivation described as the lack of intention to act on the one side and intrinsic motivation, the doing of an activity for its inherent satisfaction, on the other [51]. It can be argued that for most people, much behaviour in daily life is actually performed for external reasons. Nevertheless, it may be important whether patients follow recommended treatment just to avoid negative consequences or whether patients believe that treatment may be beneficial and will help them achieve other goals. Even if the motivation was primarily external, many participants in our study emphasized that communication and the care providers’ attitudes could make a significant difference [52]. The experience of being listened to and being involved in treatment decisions was described as a pivotal starting point for building supportive therapeutic relationships and shared treatment goals.

Collaboration with the ACT team was described as a strategy to gain increased freedom. Some participants felt the CTO was a safety net, which made it easier to be readmitted if needed. Moreover, in some situations the ACT team was obliged to intervene to prevent deterioration and serious relapse. Other participants had gradually accepted the ACT team’s medication monitoring as a buffer or as a necessary reminder to take medication to stay well. Although recognizing that the psychiatrist had the final decision making authority, several of the participants described a gradual transition toward increased influence, collaboration, and shared responsibility.

Studies from the UK and Ireland have shown that assertive outreach teams appear to be more successful than mental health care teams in engaging reluctant patients in treatment [53, 54]. The low case load and the shared responsibility within the teams imply that assertive outreach workers have more time, and more opportunities to monitor medication use and to more actively involve patients in treatment planning decisions [53, 54].

Some studies have shown that when CTOs were combined with intensive services (ACT) for more than six months, there was a substantial decrease in hospital admissions rates, total days hospitalized, and improved rates of psychotropic medication use [22, 55]. Our study is in line with other studies that show that patients’ lives seem modestly improved under ACT [56] and that being coerced does not necessarily negatively influence patients satisfaction with treatment [57, 58]. And like Phelan and colleagues [21] find, assisted outpatient treatment works as a “package deal,” where coercion is only one of the elements that have effect.

The study’s findings are in line with some other studies that show that trusting therapeutic relationships and non-judgmental and stage-wise approaches are of great importance in engaging patients in care [46, 48, 54, 59]. As Gilbert et al. [30] find, trusting therapeutic relationships seem to modify how patients across different treatment contexts interpret care providers’ behaviour and also the restrictive interventions.

The quality of the treatment relationship and the patient’s ability to influence treatment decisions seem to be factors that influence the experience of coercive practices [28, 38, 60]. In our study, many participants described the ACT team’s enactment of the CTO as a negotiated agreement, where they had strived to find a balance between the need for safety and the patient’s autonomy. As other studies show, the perception of coercion is context dependent [61]. Several of the participants in our study described the experience of feeling gradually less coerced and more autonomous, and they illustrated that the perception of coercion may change over time [53]. And as Bonnie claims [62], sometimes it seems more appropriate to reframe leveraged community treatment based upon the patients’ negotiated consent as a “contract” rather than as “coercion.”

### Strengths and limitations

This is one of very few studies that examine patients’ perceptions of CTOs within an ACT team setting. The study gives important insights into how patients that previously have avoided or not benefitted from ordinary services experience living with CTOs. It may be difficult to reach this group of patients. Nevertheless, we were able to include 15 patients with varying opinions and perspective about being subjected to CTOs. The sampling was purposive, and while we made an effort to obtain a range of different perspectives, positive and negative, the results are not representative in a statistical sense. As some of the ACT teams had very few patients on CTOs, we were unable to recruit enough patients to analyze possible differences in patients’ perceptions between the ACT teams. In recent years, it has become clearer that service users may play an important part in designing and carrying out research on the different types of services. It is a limitation that the present study did not involve service users in designing or carrying out the research.

## Conclusions

This study showed that the participants had different perceptions of being subjected to CTOs while in ACT. Some described the CTO as a security net and as an important factor for staying well. However, other participants described the CTO as a social control mechanism and as a violation of their autonomy. Many of the participants described the ACT team as a different mental health arena from what they had known before, with another frame of interaction. Despite being legally compelled to receive treatment, many participants talked about how the ACT teams focused on addressing unmet needs, the management of future crises, and finding solutions to daily life problems. Many of the patients emphasized the help they got from the ACT teams in finding housing, getting assistance with their finances, and in reducing social isolation.
